# Association of vitamin D deficiency and VDBP gene polymorphism with the risk of AMI in a Pakistani population

**DOI:** 10.12669/pjms.336.13379

**Published:** 2017

**Authors:** Mujtaba Mubashir, Shaheena Anwar, Asal Khan Tareen, Naseema Mehboobali, Khalida Iqbal, Mohammad Perwaiz Iqbal

**Affiliations:** 1 Mujtaba Mubashir, Department of Biological and Biomedical Sciences, Aga Khan University, Karachi, Pakistan; 2 Shaheena Anwar, Department of Biological and Biomedical Sciences, Aga Khan University, Karachi, Pakistan; 3 Asal Khan Tareen, CDepartment of Biochemistry/Pathology, National Institute of Cardiovascular Diseases, Karachi, Pakistan; 4 Naseema Mehboobali, Department of Biological and Biomedical Sciences, Aga Khan University, Karachi, Pakistan; 5 Khalida Iqbal, Department of Biological and Biomedical Sciences, Aga Khan University, Karachi, Pakistan; 6 Mohammad Perwaiz Iqbal, Department of Biological and Biomedical Sciences, Aga Khan University, Karachi, Pakistan

**Keywords:** Acute myocardial infarction, Coronary heart disease, Pakistani population, Vitamin D binding protein genotypes, Vitamin D deficiency

## Abstract

**Objective:**

To investigate the relationship of vitamin D deficiency and risk of AMI in a Pakistani population, and to find out any association between vitamin D binding protein (VDBP) genotypes and risk of AMI in this population.

**Methods:**

In a comparative cross-sectional study, 246 patients (age: 20-70 years; 171 males and 75 females) with first AMI were enrolled with informed consent. Similarly, 345 healthy adults (230 males and 115 females) were enrolled as controls. Their fasting serum samples were analyzed for 25 (OH) vitamin D, lipids and other biomarkers using kit methods, while DNA was analyzed for VDBP genotypes using PCR-RFLP based methods. Chi-squared test and logistic regression were used for association of vitamin D deficiency and VDBP genotypes with AMI.

**Results:**

Mean serum concentration of 25(OH) vitamin D was significantly lower in AMI patients compared to healthy subjects (p=0.015) and percent vitamin D deficiency was higher in AMI patients compared to healthy subjects (p=0.003). VDBP IF-IF genotype was positively associated with the risk of AMI in subject above 45 years after adjusting for potential confounders [OR = 9.86; 95% CI=1.16 to 83.43].

**Conclusion:**

Vitamin D deficiency and VDBP IF-IF genotype are associated with AMI in Pakistani adults.

## INTRODUCTION

Cardiovascular disease (CVD) is highly prevalent in South Asia, and is one of the leading causes of mortality. South Asians tend to develop myocardial infarction (MI) at a younger age compared to other countries, and the mortality rates due to MI are expected to continue rising in the future.^1^

Vitamin D deficiency has been shown to be an independent risk factor for CVD, with the risk varying inversely with the serum levels of vitamin D.^2^-^4^ However, this association varies with race and may not be generalizable to all ethnic groups.^5^ Vitamin D deficiency is highly prevalent in Pakistan, with around 85% of the population having insufficient or deficient serum vitamin D levels.^6^,^7^ However, except for one small study, there has been hardly any report on association of vitamin D deficiency and the risk of acute myocardial infarction (AMI) in the Pakistani population.^8^ Therefore, it is imperative to investigate the relationship between vitamin D deficiency and risk of AMI in the Pakistani population using a large sample size.

Vitamin D is transported in blood bound mainly to vitamin D binding protein (VDBP), also known as Group-specific component (Gc). VDBP is a 55-kd protein encoded by the Gc gene, located on chromosome 4.^9^,^10^ There exist three major alleles encoding VDBP (Gc-1F, Gc-1S and Gc-2) that combine to form 6 major genotypes, namely, 1F-1F, 1F-1S, 1S-1S, 1S-2, 1F-2, and 2-2.^9^ These genotypes have variable frequencies in populations of different ethnic backgrounds and have different affinities for binding vitamin D in the circulation.^9^

Few studies have explored the role of VDBP genotypes as potential risk modifiers of coronary heart disease (CHD). Some of these studies revealed no effect of the VDBP genotype distribution on the risk of developing CHD in some of the Western populations.^11^,^12^ However, no studies have been carried out on South Asian population to investigate this relationship.

Our study aimed, firstly, to identify whether an association exists between serum levels of vitamin D and the risk of developing AMI in a Pakistani population, and secondly, whether there is an association between VDBP genotypes and risk of developing AMI in a hospital based Pakistani population.

## METHODS

### Participants

591 individuals were enrolled (with informed consent) into this comparative cross-sectional study of which 246 were patients who had recently suffered an AMI and 345 were healthy subjects. The AMI patients were selected from the National Institute of Cardiovascular Diseases (NICVD) Karachi from September 2011 to December 2014. They were diagnosed with AMI as per the revised WHO classification.^13^ Subjects were excluded from the study if they were less than 20 years or more than 70 years old, if they had taken vitamin D supplementation during the last six months, if they were pregnant or suffering from chronic diseases including uremia, cancer and liver disease. The healthy subjects were selected from the staff of the Aga Khan University (AKU) and NICVD based on the same criteria and functioned as controls. This study was approved by the Ethics Review Committee of AKU.

### Measurement of serum biomarkers

Serum levels of 25(OH) vitamin D [25(OH)D], lipids, calcium, phosphate, and alkaline phosphatase (ALP) of the enrolled participants were determined using an automated kit method (Elecsys, Roche Diagnostics, Indianapolis). The serum 25(OH) D levels were then categorized as deficient (< 20 ng/ml), insufficient (20-29.9 ng/ml) or sufficient (≥ 30 ng/ml).

### Genotyping

DNA for genotyping was extracted from white blood cells obtained from the study subjects using a standard salting out method. Polymerase Chain Reaction (PCR) was used to amplify the region of interest of the VDBP gene using the method of Ito et al that has been described in detail in a previous publication.^14^,^15^

The resulting PCR product (462 bp) was digested separately with the restriction enzymes, HaeIII and Styl-HF following the principle of Restriction Fragment Length Polymorphism (RFLP). The digested PCR products were then run on a 3% agarose gel containing ethidium bromide, and VDBP genotypes were visualized as described previously.^15^

### Statistical analysis

Baseline characteristics and mean serum vitamin D levels of both the AMI patients and healthy controls were reported as mean ± SD and interquartile ranges, and compared using independent sample t-test. Frequencies of VDBP genotypes were tabulated and the Hardy Weinberg model was used to determine the frequencies of the corresponding alleles and diplotypes. Chi-squared test was used to compare vitamin D status in AMI patients and healthy subjects groups. Multiple logistic regression was used to identify any association between VDBP genotypes and the risk of developing AMI, using the most common genotype (1S-1S) as reference value. The model was adjusted for age, gender, BMI, serum LDL and vitamin D levels in order to account for potential confounders. SPSS (version 19) was used for all data analyses and p≤0.05 was considered significant.

## RESULTS

The baseline serum levels of cholesterol, LDL, HDL, ALP and calcium differed significantly between the AMI patients and healthy controls (Table-I). Moreover, 28% of AMI patients were suffering from type 2 diabetes mellitus, while none of the healthy controls had it. Nearly half of the AMI patients (46%) had hypertension, while only 4% of the controls suffered from this condition. Mean serum concentration of 25(OH) D was 13.5±14.0 ng/ml in AMI patients which was significantly lower than the mean concentration 16.1±11.7 ng/ml in healthy subjects (p=0.015). Vitamin D deficiency (<20 ng/ml) was highly prevalent among the study participants in both the groups with 83.0% of the AMI patients and 70.6% of the healthy controls classified as vitamin D deficient (Table-II). There was an association between vitamin D deficiency and AMI in this population (p=0.003).

**Table-I:** Baseline demographics, and serum biomarker concentrations in AMI patients and healthy subjects.

Characteristics*	*(n=246)*	*(n=345)*	p-value**
Gender, n (%)			
Male	171(69.5)	230(66.7)	0.46
Female	75(30.5)	115(33.5)	0.46
	Mean ± SD/ IQR (Median)	Mean ± SD/IQR (Median)	
Age (years)	43.6±8.0/ 44(5)	42.8±7.7/43(5)	0.35
BMI (kg/m^2^)	24.8±4.2/25.0(4.8)	25.7±4.8/25.3(5.7)	0.04
Cholesterol (mg/dl)	152.4± 6.1/150(66)	173.4±37.4/171(46)	<0.01
Triglycerides (mg/dl)	163.7±84.7/145(88)	151.0±88.9/132(90)	0.13
LDL-cholesterol (mg/dl)	97.6±39.4/94(54)	108.4±33.9/107(47)	<0.01
HDL-cholesterol (mg/dl)	28.3±11.2/27(11)	38.1±8.3/37(11)	<0.01
Phosphate (mg/dl)	3.8±0.9/3.7(1.0)	3.9±2.0/3.7(0.8)	0.50
Calcium (mg/dl)	8.4±1.1/8.6(1.1)	8.9±1.2/9(1.2)	<0.01
Alkaline phosphatase (U/I)	67.1±30.1/63(31)	75.0±54.3/69.0(27)	0.05
25(OH)D (ng/ml)	13.5±14.0/10.0(11.3)	16.1±11.7/13.5(14.4)	0.015

* Data are presented as the number of patients (percentage) or Mean ± standard deviation/ Interquartile Range (Median)

** p-value was obtained by comparing mean values in two groups using Independent sample t-test

**Table-II:** Vitamin D status on the basis of mean serum concentration of 25 (OH) vitamin D in AMI patients and healthy subjects

*Vitamin D status [(25(OH)D concentration]*	*AMI patients (n=246) n (%)*	*Healthy subjects (n=345) n (%)*	p-value*
Deficient (<20 ng/ml)	203(83.0)	240(70.6)	0.003
Insufficient (20-29.9 ng/ml)	26(10.6)	66(19.4)	
Sufficient (≥30 ng/ml)	16(6.5)	34(10.0)	

* p-value was obtained using Chi-squared test comparing percentages in both the groups

### Genotyping

227 AMI patients and 300 healthy controls were genotyped. The frequency distribution of VDBP alleles, diplotypes and genotypes, shown in Table-III, was in accordance with the Hardy Weinberg equilibrium (HWE) in both the groups. In both AMI patients and healthy controls, Gc 1S-1S was found to be the most common genotype (29% & 31%), while Gc 1F-1F was the least common genotype (5% & 1%). To evaluate the association of VDBP genotypes with AMI, multiple logistic regression was performed, which revealed that the Gc1F-1F genotype was positively associated with the risk of AMI in persons above 45 years after adjusting for vitamin D levels, gender, body mass index (BMI) and LDL [odds ratio=9.86; 95% confidence interval=1.16 to 83.43; Table-IV].

**Table-III:** Frequency distribution of VDBP alleles, diplotypes and genotypes in AMI patients and healthy subjects.

*VDBP*	*AMI patients (n=227) n (π)*	*Healthy controls (n=300) n (π)*
Allele		
1F	96(0.21)	106(0.17)
1S	239(0.52)	332(0.55)
2	119(0.26)	162(0.27)
Diplotype		
Gc 1-1	124(0.55)	162(0.54)
Gc 1-2	87(0.38)	114(0.38)
Gc 2-2	16(0.07)	24(0.08)
Genotype		
1F-1F	12(0.05)	4(0.01)
1S-1S	67(0.29)	94(0.31)
2-2	16(0.07)	24(0.08)
1F-1S	45(0.20)	64(0.21)
1F-2	27(0.12)	34(0.11)
1S-2	60(0.26)	80(0.26)
HWE p^a^	0.996	0.990

a p-value was obtained using the Hardy Weinberg equilibrium

**Table IV:** Association of VDBP genotypes with risk of AMI in Pakistani adults.

*VDBP genotype*	*Crude OR (95% CI)*	*Adjusted OR† (95% CI)*
1S-1S*	1	1
1F-1F	4.20(1.30-13.6)	9.86(1.16-83.43)*
2-2	0.90(0.46-1.90)	1.1(0.50-2.42)
1F-1S	0.98(0.60-1.61)	0.93(0.51-1.70)
1F-2	1.20(0.61-2.01)	1.03(0.20-2.13)
1S-2	1.03(0.66-1.66)	1.21(0.67-2.16)

* Most common genotype taken as reference value

† Adjusted for age (<45years, >45 years), gender and vitamin D levels, BMI and LDL

***Note:*** OR is odds ratio, while CI stands for confidence interval

## DISCUSSION

Our study showed that vitamin D deficiency is highly prevalent in this Pakistani population with 75.7% of all study participants classified as vitamin D deficient. This figure is consistent with previous estimates of the prevalence of vitamin D deficiency in this region.^6^,^7^

An association was found between the vitamin D deficiency and development of AMI; vitamin D deficiency was more common in AMI patients compared to healthy controls. Recent studies in the region exploring the role of vitamin D deficiency in CVD have shown an association between severe vitamin D deficiency and the risk of AMI after adjusting for conventional risk factors,^4^,^8^ while others have shown that this risk varies inversely with serum vitamin D levels.^2^ Robinson-Cohen et al. have reported that this association varies by race, such that it is not found in Blacks and Hispanics.^5^ In a recent meta-analysis, Huang et al have concluded that 25(OH)D levels were significantly lower in MI patients in America and Asia and sufficient levels of vitamin D could be protective against the occurrence of MI.^16^ Malik et al have found vitamin D deficiency to be more severe in Indian patients with MI compared to controls.^17^ Our data add support to all these findings that this association holds true in the South Asian population, with analyses showing that sufficient blood 25(OH)D levels was a protective factor for MI in this population.^4^,^16^,^17^ Thus, low serum vitamin D level is an important but often overlooked aspect of cardiovascular disease. The possible reason for this association of vitamin D deficiency with AMI merits some discussion. Low serum levels of vitamin D often accompany high levels of parathyroid hormone which has been shown to mediate cardiovascular events, such as cardiac hypertrophy, vascular remodeling and inflammation.^18^ Moreover, lower serum levels of 25(OH) D have been found to be associated with reduced serum levels of HDL, increased triglyceride levels and elevated atherogenic index of plasma.^19^ These factors could lead to the development of AMI in hypovitaminosis D.

Our study also revealed an increased risk of AMI in individuals with the VDBP genotype Gc 1F-1F, especially in those above the age of 45 years, after adjusting for confounders. Previous studies revealed no effect of VDBP genotype distribution on the risk of CHD.^11^,^12^ These findings can be explained by the fact that these studies were conducted on non-Asian populations and thus may not apply to Asian populations due to ethnic variations in the frequency distribution of VDBP genotypes.^20^ Our study is the first large study to report the association of vitamin D deficiency and AMI in a Pakistani population. It is also unique in reporting Gc 1F-1F genotype as a risk factor for AMI, albeit in the Pakistani population. Gc 1F-1F has, however, been shown to play an important role in a number of non-communicable diseases.^21^

A number of studies have been carried out to investigate the relationship of VDBP polymorphism with vitamin D deficiency and CVD. For example in a recent review article, it has been reported that VDBP polymorphism may affect the binding affinity for vitamin D, thereby, increasing the risk of vitamin D deficiency and other CVDs.^22^ Lafi et al have also reported an association of VDBP polymorphism with increased risk of vitamin D deficiency among healthy Jordanians.^23^ On the other hand, Michos et al showed that low serum levels of 25(OH)D were associated with increased incidence of CHD among Whites, however, no interaction of 25(OH)D levels with VDBP genotypes was found in that population.^11^ Similarly, VDBP genetic variants were not found to be related with CHD in Italian population.^24^ VDBP, in addition to its relationship with CHD through vitamin D deficiency, has been shown to play a relatively direct role in the development of this disease. There are several lines of evidence in this regard and various mechanisms have been proposed. Rocchiccioli et al have reported that the plasma concentration of VDBP is significantly higher in atherosclerotic patients compared to healthy subjects.^25^ VDBP expression increases with endothelial stress, and this differential expression regulates the formation of neointima in blood vessels by altering the proliferation and migration of vascular smooth muscle cells.^26^ This is further supported by proteomics-based studies showing higher levels of VDBP in thrombi of patients with acute myocardial infarction compared to control subjects.^27^ VDBP also plays an important role in tissue injury by its conversion to macrophage-activating factor (VDBP-MAF) as well as by scavenging for vascular and extracellular actin.^27^,^28^ Macrophages play a critical role in the development of atherosclerosis and ultimately coronary heart disease.^29^ Diabetes mellitus is also closely linked to atherosclerosis, and hence studies have explored the role of VDBP expression in diabetes as well.^28^ VDBP polymorphism was associated with an increased susceptibility to type 2 diabetes mellitus in Asians, while this association did not hold true in European populations.^30^ This could be one of many factors explaining why the burden of MI and diabetes is so high in South Asian population.^1^

In the present study, we identified 12 cases which had none of the classical risk factors for CHD such as hypertension, diabetes, smoking, high BMI, dyslipidemia, history of ischemic heart disease in parents. However, when the percentages of their VDBP genotypes were compared with percentages of genotypes of those AMI patients with one or more classical risk factors using chi square test, no statistically significant difference was found (p = 0.396; data not shown) suggesting that in the absence of classical risk factors, VDBP polymorphism alone does not appear to be associated with AMI. However, conclusive evidence can only be obtained using a large sample size of AMI patients with no classical risk factor for CVD.

### Limitations of the Study

There are certain limitations of our study. The Gc1F-1F genotype is the least common genotype encountered in the Pakistani population as seen in our study and hence a larger sample size of individuals carrying this genotype needs to be studied in order to further validate our findings. Moreover, surveys need to be carried out in different parts of the country to study the effect of this genotype in ethnic backgrounds not represented in the present study.

The present study also has some strength. AMI patients were sampled from NICVD, Karachi to ensure that the study sample resembled the general population closely in terms of socioeconomic backgrounds and ethnic groups. Seasonal variations in vitamin D levels were accounted for by selecting both AMI patients and healthy controls all year across. Confounding variables were accounted for in data analysis. This study is among the very few that have explored the role of VDBP genotypes in AMI patients and adds to the body of knowledge on this subject.

In terms of public health policy, this study raises several issues. As CVD is highly prevalent in our society and remains a growing cause of morbidity and mortality, vitamin D deficiency needs to be addressed as an important modifiable risk factor that could alleviate the burden of disease. Whether this risk can be alleviated by vitamin D supplementation in deficient individuals is not yet certain, however, several clinical trials are currently underway to address this question.^31^

## References

[1] Saleem MD, Mulla J, Tahir F (2012). The rising trend of myocardial infarction in young patients in Pakistan. Singapore Med J.

[2] Kienreich K, Tomaschitz A, Verheyen N, Pieber T, Gaksch M, Grübler MR (2013). Vitamin D and cardiovascular disease. Nutrients.

[3] Wang L, Song Y, Manson JE, Pilz S, März W, Michaëlsson K (2012). Circulating 25-hydroxy-vitamin D and risk of cardiovascular disease:a meta-analysis of prospective studies. Circ Cardiovasc Qual Outcomes.

[4] Roy A, Lakshmy R, Tarik M, Tandon N, Reddy KS, Prabhakaran D (2015). Independent association of severe vitamin D deficiency as a risk of acute myocardial infarction in Indians. Indian Heart J.

[5] Robinson-Cohen C, Hoofnagle AN, Ix JH, Sachs MC, Tracy RP, Siscovick DS (2013). Racial differences in the association of serum 25-hydroxyvitamin D concentration with coronary heart disease events. JAMA.

[6] Hassan S, Muzammil SM, Jafri L, Khan AH (2015). An audit of clinical laboratory data of 25 [OH]D at Aga Khan University as reflecting vitamin D deficiency in Pakistan. J Pak Med Assoc.

[7] Riaz H, Finlayson AE, Bashir S, Hussain S, Mahmood S, Malik F (2016). Prevalence of Vitamin D deficiency in Pakistan and implications for the future. Expert Rev Clin Pharmacol.

[8] Iqbal MP, Mehboobali N, Azam I, Tareen AK (2013). Association of alkaline phosphatase with acute myocardial infarction in a population with high prevalence of hypovitaminosis D. Clin Chim Acta.

[9] Gozdzik A, Zhu J, Wong BY-L, Fu L, Cole DE, Parra EJ (2011). Association of vitamin D binding protein (VDBP) polymorphisms and serum 25(OH)D concentrations in a sample of young Canadian adults of different ancestry. J Steroid Biochem Mol Biol.

[10] Cooke NE, Haddad JG (1989). Vitamin D binding protein (Gc-globulin). Endocr Rev.

[11] Michos ED, Misialek JR, Selvin E, Folsom AR, Pankow JS, Post WS (2015). 25-hydroxyvitamin D levels, vitamin D binding protein gene polymorphisms and incident coronary heart disease among whites and blacks:The ARIC study. Atherosclerosis.

[12] Vimaleswaran KS, Power C, Hyppönen E (2014). Interaction between vitamin D receptor gene polymorphisms and 25-hydroxyvitamin D concentrations on metabolic and cardiovascular disease outcomes. Diabetes Metab.

[13] Alpert JS, Thygesen K, Antman E, Bassand JP (2000). Myocardial infarction redefined--a consensus document of The Joint European Society of Cardiology/American College of Cardiology Committee for the redefinition of myocardial infarction. J Am Coll Cardiol.

[14] Ito I, Nagai S, Hoshino Y, Muro S, Hirai T, Tsukino M (2004). Risk and severity of COPD is associated with the group-specific component of serum globulin 1F allele. Chest.

[15] Anwar S, Iqbal MP, Azam I, Habib A, Bhutta S, Soofi SB (2016). Urban and rural comparison of vitamin D status in Pakistani pregnant women and neonates. J Obstet Gynaecol.

[16] Huang J, Wang Z, Hu Z, Jiang W, Li B (2017). Association between blood vitamin D and myocardial infarction:A meta-analysis including observational studies. Clin Chim Acta.

[17] Malik S, Giri S, Madhu SV, Rathi V, Banerjee BD, Gupta N (2016). Relationship of levels of Vitamin D with flow-mediated dilatation of brachial artery in patients of myocardial infarction and healthy control:A case-control study. Indian J Endocrinol Metab.

[18] Welles CC, Whooley MA, Karumanchi SA, Hod T, Thadhani R, Berg AH (2014). Vitamin D deficiency and cardiovascular events in patients with coronary heart disease:data from Heart and Soul Study. Am J Epidemiol.

[19] Wang Y, Si S, Liu J, Wang Z, Jia H, Feng K (2016). The associations of serum lipids with vitamin D status. PLoS One.

[20] Kamboh MI, Ferrell RE (1986). Ethnic variation in vitamin D-binding protein (GC):a review of isoelectric focusing studies in human populations. Hum Genet.

[21] Malik S, Fu L, Juras DJ, Karmali M, Wong BY, Gozdzik A (2013). Common variants of the vitamin D binding protein gene and adverse health outcomes. Crit Rev Clin Lab Sci.

[22] Trehan N, Afonso L, Levine DL, Levy PD (2017). Vitamin D deficiency, supplementation and cardiovascular health. Crital Pathways in Cardiology.

[23] Lafi ZM, Irshaid YM, El-Khateeb M, Ajlouni K, Hyassat D (2015). Association of rs7041 and rs4588 polymorphisms of the vitamin D binding protein and the rs10741657 polymorphism of CYP2R1 with vitamin D status among Jordanian patients. Genet Test Mol Biomarkers.

[24] Daffara V, Verdoia M, Rolla R, Nardin M, Marino P, Bellomo G (2017). Impact of polymorphism rs7041 and rs4588 of vitamin D binding protein on the extent of coronary artery disease. Nutr Metab Cardiovasc Dis.

[25] Rocchiccioli S, Pelosi G, Rosini S, Marconi M, Viglione F, Citti L (2013). Secreted proteins from carotid endarterectomy:an untargeted approach to disclose molecular clues of plaque progression. J Transl Med.

[26] Raymond MA, Désormeaux A, Labelle A, Soulez M, Soulez G, Langelier Y (2005). Endothelial stress induces the release of vitamin D-binding protein, a novel growth factor. Biochem Biophys Res Commun.

[27] Gasparri C, Curcio A, Torella D, Gaspari M, Celi V, Salituri F (2010). Proteomics reveals high levels of vitamin D binding protein in myocardial infarction. Front Biosci (Elite Ed).

[28] Stakisaitis D, Lesauskaitė V, Girdauskaitė M, Janulionis E, Ulys A, Benetis R (2016). Investigation of Vitamin D-Binding Protein Polymorphism Impact on Coronary Artery Disease and Relationship with Longevity:Own Data and a Review. Int J Endocrinol.

[29] Moore KJ, Tabas I (2011). The Cellular Biology of Macrophages in Atherosclerosis. Cell.

[30] Wang G, Li Y, Li L, Yu F, Cui L, Ba Y (2014). Association of the vitamin D binding protein polymorphisms with the risk of type 2 diabetes mellitus:a meta-analysis. BMJ Open.

[31] Chowdhury R, Kunutsor S, Vitezova A, Oliver-Williams C, Chowdhury S, Kiefte-de-Jong JC (2014). Vitamin D and risk of cause specific death:systematic review and meta-analysis of observational cohort and randomised intervention studies. BMJ.

